# Identifying the Most Sensitive and Specific Sign and Symptom Combinations for Cholera: Results from an Analysis of Laboratory-Based Surveillance Data from Haiti, 2012–2013

**DOI:** 10.4269/ajtmh.14-0429

**Published:** 2015-04-01

**Authors:** Mentor Ali Ber Lucien, Nicolas Schaad, Maria W. Steenland, Eric D. Mintz, Rossignol Emmanuel, Nicole Freeman, Jacques Boncy, Paul Adrien, Gerard A. Joseph, Mark A. Katz

**Affiliations:** National Public Health Laboratory, Ministry of Public Health and Population, Port-au-Prince, Haiti; Division of Global Health Protection, Center for Global Health, Centers for Disease Control and Prevention, Atlanta, Georgia; Division of Foodborne, Water and Environmental Diseases, National Center for Emerging and Zoonotic Infections, Centers for Disease Control and Prevention, Atlanta, Georgia; Directorate of Epidemiology, Laboratory and Research, Ministry of Public Health and Population, Port-au-Prince, Haiti; Centers for Disease Control and Prevention—Haiti, Port-au-Prince, Haiti

## Abstract

Since October 2010, over 700,000 cholera cases have been reported in Haiti. We used data from laboratory-based surveillance for diarrhea in Haiti to evaluate the sensitivity, specificity, and positive (PPV) and negative predictive values (NPV) of the cholera case definitions recommended by the World Health Organization (WHO). From April 2012 to May 2013, we tested 1,878 samples from hospitalized patients with acute watery diarrhea; 1,178 (62.7%) yielded *Vibrio cholerae* O1. The sensitivity and specificity of the WHO case definition for cholera in an epidemic setting were 91.3% and 43.1%, respectively, and the PPV and NPV were 72.8% and 74.8%, respectively. The WHO case definition for cholera in an area where cholera is not known to be present had lower sensitivity (63.1%) and NPV (55.1%) but higher specificity (74.2%) and PPV (80.0%). When laboratory diagnostic testing is not immediately available, clinicians can evaluate signs and symptoms to more accurately identify cholera patients.

## Introduction

A cholera epidemic began in Haiti in October 2010. From October 20, 2010 to December 31, 2013, 697,392 cholera cases were reported to Haiti's National Cholera Surveillance System (NCSS).^1^ Currently in Haiti, all patients with acute watery diarrhea who are treated at cholera treatment facilities (CTFs) are reported as cases of cholera to the NCSS.^2^ Most clinics and CTFs in Haiti lack on-site capacity to confirm suspected cholera cases with laboratory diagnostics. This makes syndromic case definitions essential, both for surveillance and clinical purposes. However, cholera can be difficult to diagnose on clinical grounds alone.^3^,^4^ Results from the limited testing conducted in Haiti show that a considerable percentage of reported syndromic cholera cases have tested negative for cholera; a recent report of laboratory diagnostic testing of patients who presented to four Haitian hospitals with acute diarrhea from 2012–2013 showed that although 73% of over 5 years old with acute diarrhea had laboratory-confirmed cholera, only 26% of patients < 5 years old with acute diarrhea had laboratory-confirmed cholera.^5^ Similar work conducted in Haiti from 2011 to 2012 found that 41.8% of the patients sampled who came to a cholera treatment center and 19.8% of those seen in the community oral rehydration points had acute diarrhea caused by cholera.^6^ Similarly, during the first months of the cholera outbreak in the Dominican Republic in 2010, less than one-fifth of suspected cholera cases were positive for cholera by culture.^7^

**Figure 1.**
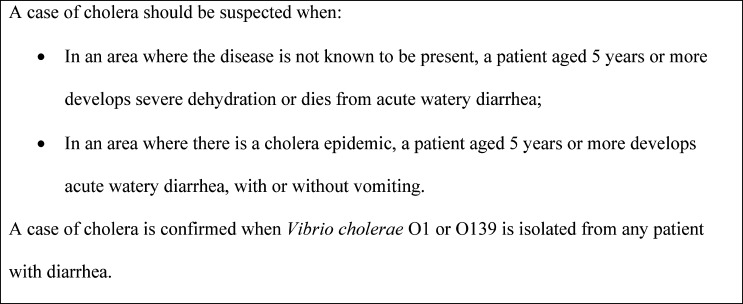
WHO standard case definitions for cholera surveillance.^8^

It has been over 4 years since the cholera epidemic began in Haiti. As the epidemic matures, having a better understanding of the accuracy of different sign and symptom combinations in accurately diagnosing laboratory-confirmed cholera is important to inform national surveillance guidelines and clinical decision making. We used data from a laboratory-based sentinel surveillance system for acute diarrheal illness in Haiti, which complements NCCS, to evaluate the sensitivity, specificity, positive predictive value (PPV), and negative predictive value (NPV) of the two case definitions recommended by World Health Organization (WHO) for cholera surveillance (Figure 1), and other combinations of clinical signs and symptoms.

## Materials and Methods

**Figure 2.**
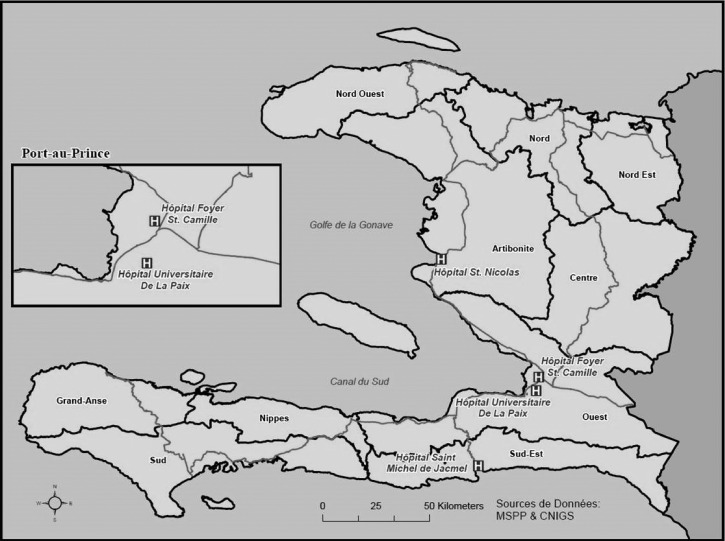
Laboratory-based sentinel surveillance sites, Haiti, 2012–2013.

We used data from laboratory-based surveillance being conducted at selected four hospitals.^5^ The first two hospitals, Hôpital Universitaire De La Paix (HUP) and Hôpital Foyer Saint Camille (HSC), are located in the capital of Haiti, Port-au-Prince, in the west department. The third site, Hôpital Saint Nicolas (HSN) de Saint Marc, is in the Artibonite Department, and the fourth, Hôpital Saint Michel de Jacmel (SMJ), is in the southeast department (Figure 2). We chose these four hospitals because they all have associated CTFs, have relatively large facilities, and are located within a 3-hour drive from Laboratoire Nationale de Sante Publique (LNSP) in Port-au-Prince, making transport of specimens manageable. At each site, trained nurses used convenience sampling to collect stool specimens from up to 10 hospitalized patients per week with acute watery diarrhea defined as three or more episodes of acute watery diarrhea within 24 hours, with onset of symptoms within the past 7 days.^5^ Patients who had taken antibiotics either at home or in the health facility were excluded. Patients were selected from CTFs, pediatric wards, medicine wards, and emergency rooms. We also administered a questionnaire to patients to collect demographic and clinical information. Nurses determined a patient's clinical symptoms, including dehydration status, by a combination of chart review, questions, and physical exam. Dehydration status was defined according to WHO case definitions (http://whqlibdoc.who.int/publications/2005/9241593180.pdf). Moderate dehydration was defined as restless or irritable behavior, poor skin turgor, rapid pulse, and moderate increase in thirst. Severe dehydration was defined as a lethargic or comatose patient with a rapid and weak pulse, very poor skin turgor, and a major increase in thirst.

Whole stool was collected in a cup, and two swabs were placed into Cary-Blair transport medium. Whole stool and inoculated transport medium were stored at 2–8°C for up to 3 days before transport to LNSP. At LNSP, specimens were tested by culture for *V. cholerae*, as described previously.^5^

### Data Analysis.

**Figure 3.**
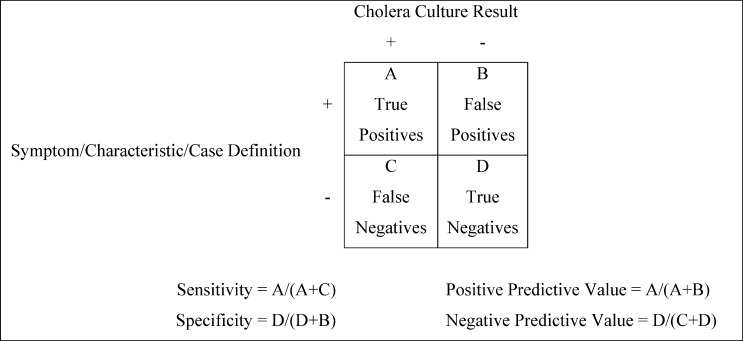
The calculation of sensitivity, specificity, positive predictive value (PPV), and negative predictive value (NPV).

Data were entered and stored in a Microsoft Access 2010 database (Microsoft Corporation, Redmond, WA) and analyzed using Epi Info 7 (U.S. Centers for Disease Control and Prevention [CDC], Atlanta, GA). Frequency procedures were used to generate descriptive statistics. Bivariate and multivariate analyses were conducted to assess whether demographic characteristics and clinical symptoms of patients were associated with culture-positive cholera. Only symptoms and characteristics known to be associated with cholera that were present in > 20% patients tested for cholera were included in the analyses. Symptoms and characteristics that were statistically significant were included in a multivariable model, and two-way interactions were assessed. We then evaluated the sensitivity, specificity, PPV, and PPV of the two WHO case definitions and additional combinations of demographic characteristics and clinical symptoms using cholera culture as the gold standard (Figure 3). The various case definitions were evaluated for patients of all ages, as well as patients under 5 years.

Verbal consent was obtained from adults and from the parents or guardians of minors. The verbal consent procedures and the rest of the activities described in this evaluation were approved as part of a non-research surveillance activity by both the CDC Institutional Review Board and the Haiti National Bioethics Committee.

## Results

### Demographic characteristics.

From April 2, 2012 to May 14, 2013, a total of 1,878 stool samples were collected from patients presenting with acute watery diarrhea at the four sentinel sites (Table 1). Almost half of sampled patients were female (47.7%). The median age was 29 years, and most patients (78.4%) were aged 5 years or older. Of the 1,878 samples tested by culture, 1,178 (62.7%) yielded *V. cholerae*. A higher percentage of patients over 5 years (72.8%) tested positive for cholera compared with patients under 5 years (25.2%).

### Clinical symptoms and cholera.

Bivariate analyses revealed a significant association between age ≥ 5 years and culture-positive cholera (odds ratio [OR]: 8.0, 95% confidence interval [CI]: 6.2–10.3) (Table 2). Moderate dehydration alone was not significantly associated with culture-positive cholera (OR: 1.0, 95% CI: 0.5–1.7), however severe dehydration was (OR: 3.0, 95% CI: 1.7–5.3). Muscle pain (OR: 1.6, 95% CI: 1.3–2.0), nausea (OR: 1.9, 95% CI: 1.5–2.3), and vomiting (OR: 3.3, 95% CI: 2.6–4.3) were all significantly associated with cholera. In terms of the number of stools in the past 24 hours, patients with 10–14 (OR: 5.2, 95% CI: 3.0–9.2), 15–19 (OR: 5.2, 95% CI: 2.9–9.3), 20–24 (OR: 12.2, 95% CI: 6.5–22.9), and 25 or more stools (OR: 10.3, 95% CI: 5.3–19.9) had significantly higher odds of having culture-positive cholera compared with patients who had 3–4 stools in the past 24 hours. Neither abdominal pain nor the number of days of diarrhea was significantly associated with culture-positive cholera. With the exception of nausea and muscle pain, all symptoms that were significant in bivariate analyses remained significant in multivariate analyses.

### Sensitivity, specificity, PPV, and NPV of case definitions.

The sensitivity and specificity of the WHO case definition for cholera in an epidemic setting (a patient aged 5 years or more develops acute watery diarrhea, with or without vomiting) were 91.3% (95% CI: 89.6–92.8) and 43.1% (95% CI: 39.5–46.8), respectively, and the PPV and NPV were 72.8% (95% CI: 70.5–76.1) and 74.8% (95% CI: 70.3–78.8), respectively (Table 3).^8^ The WHO case definition for cholera in an area where the disease is not known to be present (a patient aged 5 years or more develops severe dehydration or dies from acute watery diarrhea) had a lower sensitivity (63.1%, 95% CI: 60.1–66.0) and NPV (55.1%, 95% CI: 51.8–58.5) but a higher specificity (74.2%, 95% CI: 70.7–77.5) and PPV (80.0%, 95% CI: 77.1–82.7). When we added moderate dehydration to the latter WHO case definition, the sensitivity and specificity were 88.7% (95% CI: 86.6–90.5) and 47.7% (95% CI: 43.8–51.6), respectively.

Of all the combinations we explored among patients of all ages, modifying the current syndromic case definition to include only cases ≥ 5 years old (the recommended WHO case definition for cholera epidemic areas) achieved a very high sensitivity for cholera (91.3%). Further restricting the definition to include only cases ≥ 5 years old with moderate to severe diarrhea only minimally decreased the sensitivity to 88.7%.^8^ In contrast, adding muscle pain, nausea, vomiting, increasing the number of stools in 24 hours, and limiting the case definition to severely dehydrated patients reduced the sensitivity of the case definition considerably but also increased specificity. The combination of age ≥ 5 years, severe dehydration, and an increased (≥ 25) number of stools per 24 hours achieved the highest specificity (99.5%, 95% CI: 98.3–99.9) and PPV (92.0%, 95% CI: 75.0–97.8).

Among patients under 5 years, a case definition including all patients with moderate or severe dehydration achieved the highest sensitivity (98.9, 95% CI: 94.1–99.8) (Table 4). Similar to results for patients of all ages, the addition of vomiting, nausea, or muscle pain or increasing the threshold for the number of stools in the last 24 hours led to lower sensitivities but higher specificities. The inclusion of muscle pain or a high number of stools in particular led to high specificities and PPVs.

## Discussion

To the best of our knowledge this is the first study to look at the statistical measures of performance of different combinations of demographic characteristics, clinical signs, and symptoms, including the case definitions currently recommended by WHO, for cholera surveillance.^8^ A similar assessment has been carried out with regards to the WHO-recommended case definition for typhoid fever.^9^ In Haiti, where cholera has circulated widely since October 2010 and in other countries where laboratory testing of every suspected cholera case is either impractical or inefficient, our findings can help inform decisions about how to optimize syndromic cholera case definitions to meet surveillance and clinical objectives.

We found that the WHO case definition for cholera in an epidemic setting demonstrated a high sensitivity and a moderate specificity. If the goal were to obtain a more specific case definition than the one currently used by NCSS in Haiti, this case definition could be an option. Although the highly significant association between age group and cholera status that was found in our bivariate analyses provides strong support for the exclusion of children < 5 years old from the case definitions recommended by WHO, such a switch to this case definition would certainly lead to missed cholera cases. The current case definition for cholera in Haiti (a patient of any age with acute watery diarrhea) likely falsely classifies many diarrhea cases as cholera, particularly among children < 5 years old.^5^ Nonetheless, in our four sites, 25.2% children < 5 years old with acute diarrhea had culture-confirmed cholera. NCSS has collected information on the age group (≥ 5 versus < 5 years old) of reported cholera patients since the beginning of the epidemic.^2^ As of December 31, 2013, children < 5 years old had accounted for 13.5% of 697,392 total reported cholera cases. This age group continues to be susceptible to cholera in Haiti, and as Haiti transitions from an epidemic pattern to a more endemic pattern of cholera transmission, young children with no previous exposure or prior immunity may eventually comprise an even greater proportion of all cholera hospitalizations, as occurs in other countries.^10^,^11^

We found that a number of sign and symptom combinations could provide higher levels of specificity for cholera surveillance in Haiti. However, as expected, our findings also show that modifying the current case definition or those recommended by WHO to a more specific one would come at the price of sensitivity. For an epidemic-prone disease such as cholera, a surveillance system with low sensitivity could fail to detect small outbreaks, and potentially delay mobilization of life-saving clinical resources such as IV fluids and preventive measures. Changing a surveillance case definition can have important methodological and political implications. For example, the apparent reduction in the number of cases that would occur with a switch to the WHO case definition for epidemic areas could lead to a false sense of security or reduced resources for response and prevention efforts. In addition, such a change would complicate the comparison of new data with historic data and analyses of trends across time.

Beyond cholera surveillance, the results of our analysis could potentially provide further guidance for clinicians who treat suspected cholera cases without laboratory diagnostic tools. We found an association between several symptoms—muscle pain, nausea, vomiting, and the number of stools in the past 24 hours—and culture-positive cholera among diarrhea patients. These clinical symptoms are typical of cholera and have been described previously.^12^ Our findings related to the PPV and NPV of different sign and symptom combinations could be especially useful in areas where treatment resources are limited. Ultimately, the treatment of patients with diarrhea depends largely on the degree of dehydration and not the etiology. However, there are scenarios in which a better understanding of the etiology could prove useful. For example, antimicrobial treatment is recommended for cholera patients with severe dehydration but not for rotavirus patients with severe dehydration.^13^

Our findings should be considered in light of the fact that sensitivity, specificity, PPV, and NPV can vary with disease prevalence in the population.^14^ In Haiti, cholera activity decreased in prevalence over the first three full years of the epidemic: as of December 31, 2013 only 58,391 syndromic cases had been reported, compared with 101,503 in 2012 and 352,033 in 2011.^2^ In other countries where cholera is endemic, prevalence can also vary considerably. In Bangladesh, cholera can be undetectable at certain times of the year, and in many countries the magnitude of cholera epidemics varies dramatically from year to year.^15^,^16^ Therefore, although our findings may be applicable to other settings, they should be interpreted in the context of the relevant prevalence of cholera.

Our findings are subject to several limitations. First, stool samples were only collected from patients at four hospitals in three departments, which limits the generalizability of the results to the rest of the country. Second, stool samples were collected from a convenience sample of hospitalized patients. Only 55% of all cholera case reported to NCSS since the beginning of the Haiti cholera epidemic were hospitalized.^2^ Our findings may not be applicable to outpatients. Although all the four hospitals and their associated CTFs were located in central locations within cities, it is possible that patients with severe disease located further away from the hospitals would have had more difficulty accessing the hospitals or CTFs, which may have biased our results toward patients who lived closer to the hospital. In addition, although we collected extensive demographic and clinical information in our questionnaire, there were some signs, such as rice-water stool, that were omitted. This and other signs should be evaluated in future studies. In addition, although all nurses were trained on identifying signs and symptoms associated with dehydration status, classification may have varied by individual nurses. We also only collected samples from patients with three or more episodes of acute watery diarrhea within 24 hours. However, over 70% infected persons can be asymptomatic, and would not have been captured by this surveillance system; and therefore our results are not applicable to asymptomatic cholera cases or cholera cases with fewer than three episodes of diarrhea in a day.^17^,^18^ In addition, although we instructed surveillance officers to do the best they could to evaluate whether the medications the patients said they had taken were in fact antibiotics, which are easily available in Haiti outside the hospital, it is possible that a small number of patients who took antibiotics were included, and the inverse is possible as well. Finally, although we used stool culture, the recognized gold standard for cholera detection, we were not able to test specimens by polymerase chain reaction (PCR).^19^ Although there is no current consensus on a single, validated PCR method for cholera diagnosis, the combined use of stool culture and PCR in future studies could increase the sensitivity of cholera detection.^20^

More than 4 years after the beginning of the epidemic, cholera remains an important public health issue in Haiti. Improving access to safe drinking water and sanitation is the best long-term solution to reducing morbidity and mortality because of cholera and other diarrheal diseases. Oral cholera vaccines may also have a role in cholera prevention and control in Haiti as a complement to more traditional measures. In parallel, rigorous surveillance coupled with timely outbreak response and effective clinical management can contribute to saving lives. A simple, standardized case definition is central to these efforts. No single case definition is perfect; public health practitioners and policymakers must strike a balance between sensitivity and specificity according to the objectives of the surveillance system, which may change as cholera epidemics run their course over time. Although our work highlights several potential alternative case definitions for cholera surveillance in Haiti, none stand out as a clear-cut, consensus alternative, especially when weighing the practical and political implications of modifying a case definition during an ongoing epidemic. Nonetheless, our results can contribute to improving the understanding of the dynamics of the cholera epidemic in Haiti.

## Figures and Tables

**Table 1:** Characteristics of hospitalized patients tested for *Vibrio cholerae*, Haiti, 2012–2013*

Patient characteristic	Total tested for cholera *N* (%)
Age	
< 5 years	401 (21.6)
≥ 5 years	1458 (78.4)
Sex	
Male	981 (52.4)
Female	893 (47.7)
Surveillance site	
Hôpital Universitaire de la Paix	673 (35.8)
Hôpital Foyer Saint-Camille	398 (21.2)
Hôpital Saint-Nicolas	440 (23.4)
Hôpital Saint-Michel de Jacmel	367 (19.5)
Total	1878 (100.0)

* Missing values excluded.

**Table 2:** Bivariate analyses of clinical symptoms and characteristics of hospitalized patients tested for *Vibrio cholerae*, Haiti, 2012–2013*

Characteristic/symptom	Cholera positive *N* (%)	Cholera negative *N* (%)	OR	95% CI	*P* value
Age group					
< 5 years	101 (72.8)	300 (27.2)		Ref.	
≥ 5 years	1062 (25.2)	396 (74.8)	8.0	6.2–10.3	< 0.01
Dehydration					
No dehydration	25 (48.1)	27 (51.9)		Ref.	
Moderate	308 (47.0)	347 (53.0)	1.0	0.5–1.7	0.88
Severe	695 (73.7)	248 (26.3)	3.0	1.7–5.3	< 0.01
Abdominal pain					
Yes	856 (67.2)	417 (32.8)	1.2	1.0–1.5	0.11
No	309 (63.2)	180 (36.8)		Ref.	
Muscle pain					
Yes	317 (74.4)	109 (25.6)	1.6	1.3–2.0	< 0.01
No	842 (64.6)	461 (35.4)		Ref.	
Nausea					
Yes	807 (70.4)	339 (29.6)	1.9	1.5–2.3	< 0.01
No	359 (56.1)	281 (43.9)		Ref.	
Vomiting					
Yes	1045 (68.2)	487 (31.8)	3.3	2.6–4.3	< 0.01
No	133 (39.1)	207 (60.9)		Ref.	
Days of diarrhea					
1–2	638 (63.9)	361 (36.1)		Ref.	
3–5	365 (60.9)	234 (39.1)	0.9	0.7–1.1	0.24
6+	31 (58.5)	22 (41.5)	0.8	0.5–1.4	0.43
Number of stools in last 24 hours					
3–4	21 (29.6)	50 (70.4)		Ref.	
5–9	151 (41.5)	213 (58.5)	1.7	1.0–2.9	0.06
10–14	191 (68.7)	87 (31.3)	5.2	3.0–9.2	< 0.01
15–19	150 (68.5)	69 (31.5)	5.2	2.9–9.3	< 0.01
20–24	169 (83.7)	33 (16.3)	12.2	6.5–22.9	< 0.01
25+	112 (81.2)	26 (18.8)	10.3	5.3–19.9	< 0.01

* Missing values excluded.

**Table 3:** Sensitivity, specificity, PPV, and NPV of clinical case definitions for cholera, Haiti, 2012–2013*

Case definition†	No. of patients in cell‡				Sensitivity % (95% CI)	Specificity % (95% CI)	PPV % (95% CI)	NPV % (95% CI)
	A	B	C	D				
WHO case definitions								
Age ≥ 5 years	1062	396	101	300	91.3 (89.6–92.8)	43.1 (39.5–46.8)	72.8 (70.5–75.1)	74.8 (70.3–78.8)
Age ≥ 5 years and severe dehydration	641	160	375	461	63.1 (60.1–66.0)	74.2 (70.7–77.5)	80.0 (77.1–82.7)	55.1 (51.8–58.5)
Moderate and severe dehydration								
Severe dehydration	695	248	333	374	67.6 (64.7–70.4)	60.3 (56.2–63.9)	73.7 (70.8–76.4)	52.9 (49.2–56.6)
Moderate or severe dehydration	1003	595	25	27	97.6 (96.4–98.4)	4.3 (3.0–6.2)	62.8 (60.4–65.1)	51.9 (38.7–64.9)
Age ≥ 5 years and moderate or severe dehydration	901	325	115	296	88.7 (86.6–90.5)	47.7 (43.8–51.6)	73.5 (71.0–75.9)	72.0 (67.5–76.1)
Nausea								
Nausea	807	339	359	281	69.2 (66.5–71.8)	45.3 (41.4–49.3)	70.4 (67.7–73.0)	43.9 (40.1–47.8
Age ≥ 5 years and nausea	738	250	413	367	64.1 (61.3–66.8)	59.5 (55.6–63.3)	74.7 (71.9–77.3)	47.1 (43.5–50.6)
Age ≥ 5 years and severe dehydration and nausea	458	107	547	440	45.6 (42.5–48.7)	80.4 (76.9–83.6)	81.1 (77.6–84.1)	44.6 (41.5–47.7)
Vomiting								
Vomiting	1045	487	133	207	88.7 (86.8–90.4)	29.8 (26.5–33.3)	68.2 (65.8–70.5)	60.9 (55.6–65.9)
Age ≥ 5 years and vomiting	947	308	216	383	81.4 (79.1–83.6)	55.4 (51.7–59.1)	75.5 (73.0–77.8)	63.9 (60.0–67.7)
Age ≥ 5 years and severe dehydration and vomiting	592	140	424	478	58.3 (55.2–61.3)	77.4 (73.9–80.5)	80.9 (77.9–83.6)	53.0 (49.7–56.2)
Muscle pain								
Muscle pain	317	109	842	461	27.4 (24.9–30.0)	80.9 (77.5–83.9)	74.4 (70.1–78.3)	35.4 (32.8–38.0)
Age ≥ 5 years and muscle pain	294	96	850	472	25.7 (23.3–28.3)	83.1 (79.8–86.0)	75.4 (70.9–79.4)	35.7 (33.2–38.3)
Age ≥ 5 years and severe dehydration and muscle pain	140	41	860	465	14.0 (12.0–16.3)	91.9 (89.2–94.0)	77.4 (70.7–82.8)	35.1 (32.6–37.7)
Number of stools in last 24 hours								
≥ 5 stools	773	428	21	50	97.4 (96.0–98.3)	10.5 (8.0–13.5)	64.4 (61.6–7.0)	70.4 (59.0–79.8)
≥ 10 stools	622	215	172	263	78.3 (75.3–81.1)	55.0 (50.5–59.4)	74.3 (71.2–77.2)	60.5 (55.8–64.9)
≥ 15 stools	431	128	363	350	54.3 (50.8–57.7)	73.2 (69.1–77.0)	77.1 (73.4–80.4)	49.1 (45.4–52.8)
≥ 20 stools	281	59	513	419	35.4 (32.1–38.8)	87.7 (84.4–90.3)	82.7 (78.3–86.3)	45.0 (41.8–48.2)
≥ 25 stools	112	26	682	452	14.1 (11.9–16.7)	94.6 (92.1–96.2)	81.2 (73.8–86.8)	39.9 (37.1–42.7)
Age ≥ 5 years and ≥ 5 stools	698	253	90	223	88.6 (86.2–90.6)	46.9 (42.4–51.3)	73.4 (70.5–76.1)	71.3 (66.0–76.0)
Age ≥ 5 years and ≥ 10 stools	580	171	208	305	73.6 (70.5–76.6)	64.1 (59.7–68.3)	77.2 (74.1–80.1)	59.5 (55.2–63.6)
Age ≥ 5 years and ≥ 15 stools	406	107	382	369	51.5 (48.0–55.0)	77.5 (73.6–81.0)	79.4 (75.4–82.4)	49.3 (45.6–52.7)
Age ≥ 5 years and ≥ 20 stools	268	51	520	425	34.0 (30.8–37.4)	89.3 (86.2–91.8)	84.0 (79.6–87.6)	45.0 (41.8–48.2)
Age ≥ 5 years and ≥ 25 stools	105	23	683	453	13.3 (11.1–15.9)	95.2 (92.9–96.8)	82.0 (74.5–87.7)	39.9 (37.1–42.8)
Age ≥ 5 years and severe dehydration and ≥ 5 stools	423	100	251	323	62.8 (59.1–66.3)	76.4 (72.1–80.2)	80.9 (77.3–84.0)	56.3 (52.2–60.3)
Age ≥ 5 years and severe dehydration and ≥ 10 stools	355	76	319	347	52.7 (48.9–56.4)	82.0 (78.1–85.4)	82.4 (78.5–85.7)	52.1 (48.3–55.9)
Age ≥ 5 years and severe dehydration and ≥ 15 stools	215	44	459	379	31.9 (28.5–35.5)	89.6 (86.3–92.2)	83.0 (78.0–87.1)	45.2 (41.9–48.6)
Age ≥ 5 years and severe dehydration and ≥ 20 stools	122	19	552	404	18.1 (15.4–21.2)	95.5 (93.1–97.1)	86.5 (79.9–91.2)	42.3 (39.2–45.4)
Age ≥ 5 years and severe dehydration and ≥ 25 stools	23	2	651	423	3.4 (2.3–5.1)	99.5 (98.3–99.9)	92.0 (75.0–97.8)	39.4 (36.5–42.3)

NPV = negative predictive value; PPV = positive predictive value.

* Missing values excluded.

† All case definitions include acute watery diarrhea.

‡ Refer to Figure 3.

**Table 4:** Sensitivity, specificity, PPV, and NPV of clinical case definitions for cholera among children under 5 years, Haiti, 2012–2013*

Case definition†	No. of patients in cell‡				Sensitivity % (95% CI)	Specificity % (95% CI)	PPV % (95% CI)	NPV % (95% CI)
	A	B	C	D				
Moderate and severe dehydration								
Severe dehydration	48	88	44	189	52.2 (42.1–62.1)	68.2 (62.5–73.4)	35.3 (27.8–43.6)	81.1 (75.6–85.6)
Mod. or severe dehydration	91	269	1	8	98.9 (94.1–99.8)	2.9 (1.5–5.6)	25.3 (21.1–30.0)	88.9 (56.5–98.0)
Nausea								
Nausea	60	86	34	145	63.8 (53.8–72.8)	62.8 (56.4–68.8)	41.1 (33.4–49.2)	81.0 (74.6–86.1)
Severe dehydration and nausea	26	33	59	178	30.6 (21.8–41.1)	84.4 (78.9–88.6)	44.1 (32.2–56.7)	75.1 (69.2–80.2)
Vomiting								
Vomiting	86	176	15	122	85.2 (76.9–90.8)	22.5 (17.5–28.3)	32.8 (27.4–38.7)	77.3 (65.8–85.7)
Severe dehydration and vomiting	41	62	51	214	44.6 (34.8–54.7)	77.5 (72.3–82.1)	39.8 (30.9–49.5)	80.8 (75.6–85.1
Muscle pain								
Muscle pain	15	12	70	171	17.7 (11.0–27.1)	93.4 (88.9–96.2)	55.6 (37.3–72.4)	71.0 (64.9–76.3)
Severe dehydration and muscle pain	11	6	66	166	14.3 (8.2–23.8)	96.5 (92.6–98.4)	64.7 (41.3–82.7)	71.6 (65.4–77.0)
Number of stools in last 24 hours								
≥ 5 stools	69	173	7	36	90.8 (82.2–95.5)	17.2 (12.7–22.9)	28.5 (23.2–34.5)	83.7 (70.0–91.9)
≥ 10 stools	38	44	38	165	50.0 (39.0–61.0)	78.9 (72.9–83.9)	46.3 (36.0–57.1)	81.3 (75.4–86.1)
≥ 15 stools	23	21	53	188	30.3 (21.1–41.3)	90.0 (85.1–93.3)	52.3 (37.9–66.3)	78.0 (72.4–82.8)
≥ 20 stools	11	8	65	201	14.5 (8.3–24.1)	96.2 (92.6–98.1)	57.9 (36.3–76.9)	75.6 (70.1–80.3)
≥ 25 stools	6	3	70	206	7.9 (3.7–16.2)	98.6 (95.9–99.5)	66.7 (35.4–87.9)	74.6 (69.2–79.4)
Severe dehydration and ≥ 5 stools	26	50	41	145	38.8 (28.1–50.8)	74.4 (67.8–80.0)	34.2 (24.5–45.4)	78.0 (71.5–83.3)
Severe dehydration and ≥ 10 stools	13	21	54	174	19.4 (11.7–30.4)	89.2 (84.1–92.9)	38.2 (23.9–55.0)	76.3 (70.4–81.4)
Severe dehydration and ≥ 15 stools	7	9	60	186	10.5 (5.2–20.0)	95.4 (91.5–97.6)	43.8 (23.1–66.8)	75.6 (69.9–80.6)
Severe dehydration and ≥ 20 stools	5	4	62	191	7.5 (3.2–16.3)	98.0 (94.9–99.2)	55.6 (26.7–81.1)	75.5 (69.8–80.4)
Severe dehydration and ≥ 25 stools	2	0	65	195	3.0 (0.8–10.3)	100.0 (98.1–100.1)	100.0 (34.2–100.0)	75.0 (69.4–79.9)

NPV = negative predictive value; PPV = positive predictive value.

* Missing values excluded.

† All case definitions include acute watery diarrhea.

‡ Refer to Figure 3.
